# Databases for multilevel biophysiology research available at Physiome.jp

**DOI:** 10.3389/fphys.2015.00251

**Published:** 2015-09-09

**Authors:** Yoshiyuki Asai, Takeshi Abe, Li Li, Hideki Oka, Taishin Nomura, Hiroaki Kitano

**Affiliations:** ^1^Integrated Open Systems Unit, Okinawa Institute of Science and Technology Graduate UniversityOkinawa, Japan; ^2^Intasect Communications Inc.Osaka, Japan; ^3^Neuroinformatics Japan Center, RIKEN Brain Science InstituteSaitama, Japan; ^4^Department of Mechanical Science and Bioengineering, Osaka UniversityOsaka, Japan; ^5^The Systems Biology InstituteTokyo, Japan

**Keywords:** databases, physiome and systems biology, multilevel models, model sharing, team development, interoperability among databases, interoperability with applications

## Abstract

Physiome.jp (http://physiome.jp) is a portal site inaugurated in 2007 to support model-based research in physiome and systems biology. At Physiome.jp, several tools and databases are available to support construction of physiological, multi-hierarchical, large-scale models. There are three databases in Physiome.jp, housing mathematical models, morphological data, and time-series data. In late 2013, the site was fully renovated, and in May 2015, new functions were implemented to provide information infrastructure to support collaborative activities for developing models and performing simulations within the database framework. This article describes updates to the databases implemented since 2013, including cooperation among the three databases, interactive model browsing, user management, version management of models, management of parameter sets, and interoperability with applications.

## 1. Introduction

Recently, large quantities of experimental physiological data, from the macromolecular and cellular levels to the organ and organismal levels, have been accumulating in the public domain. To understand biophysiological phenomena at the system level, mathematical models representing detailed physiological functions based on those data have become increasingly important. Given their potential applications to predictive medicine and pharmaceutical science, such models must become more precise and sophisticated, and accordingly, larger in size.

The process of building detailed models can be traced to the inauguration of the Physiome Project, which was introduced conceptually at the Commission on Bioengineering of the International Union of Physiological Sciences in 1993 (Hunter et al., 2002). The first step of the Physiome Project was to establish an encyclopedic database of physiological functions (Bassingthwaighte, 2000). Physiological systems to be modeled are so huge that it is impossible for a single research group to model them. Hence, it is not only necessary to share data, but also to facilitate collaborations among research groups doing both wet and dry research.

At approximately the same time, the concept of systems biology was proposed. This is a research strategy to investigate and understand organismal biology as a system, and to focus on dynamics of networks of biological functions (Kitano, 2002a,b). Because of the nonlinearity of biological systems, applications such as estimating the dynamics of medicinal effects or predicting the toxicity of pharmaceuticals, are extremely complex. The systems biology approach emphasizes the importance of building detailed mathematical models to apply simulations to predictive medicine.

In addition to databases, it is also necessary to establish software frameworks that share and reuse existing models to develop detailed biophysiological models. The importance of technological developments in facilitating these research streams has been widely recognized, and several projects, such as Physiome Project (http://www.physiome.org) and the IUPS Physiome Project (http://physiomeproject.org), have been promoted to develop necessary tools.

Regarding model development support, several model description formats based on XML have been proposed, such as Systems Biology Markup Language (SBML) (Hucka et al., 2003), which excels at describing intracellular phenomena, CellML (Lloyd et al., 2004), which describes physiological phenomena, and NeuroML (Goddard et al., 2001), which is specialized for modeling the nervous system.

There are model databases and repositories of models written in these formats. For example, BioModels (http://www.ebi.ac.uk/biomodels-main/) (Le Novère et al., 2006; Chelliah et al., 2014) includes and publishes many computable open-domain SBML models. Those are divided into two categories, curated and non-curated. Curated models are certified as capable of being simulated. Models written in CellML are listed in the CellML Repository (https://models.physiomeproject.org) (Lloyd et al., 2008). Models related to the nervous system, some of which are written in NeuroML, are stored in Open Source Brain (http://www.opensourcebrain.org) and ModelDB (http://senselab.med.yale.edu/modeldb/) at Yale University. Such standardized formats and model databases have encouraged sharing of models.

Another effort to develop a description language is called Physiological Hierarchy Markup Language (PHML). This is a successor to *insilico*ML (ISML) (Asai et al., 2008), and is also an XML-based model description format designed to explicitly describe network and hierarchical structures seen in physiological functions. PHML has partial compatibility with CellML (Suzuki et al., 2008), i.e., most features of CellML can be translated into PHML, and PHML has some extra capabilities for describing models that are complementary to those of CellML.

The PHML model database is accessible at Physiome.jp, which was established by a MEXT global COE project “an *in-silico*, medicine-oriented, worldwide open platform,” led by one of the authors (TN) in 2007. The project sought to develop the infrastructure for predictive medicine based on collaborations among physicians, engineers, and informaticians (Nomura, 2010). It is freely available at http://physiome.jp. In parallel with the model database, Physiome.jp also maintains two other databases containing morphological and time-series data, in order to manage models and associated data comprehensively. In addition to databases, tools to support construction of multi-hierarchical, large-scale, physiological models are also offered there.

Applications available at Physiome.jp include a modeling platform named PhysioDesigner, a simulator called Flint, and PhysioVisualizer. The latter visualizes simulation results generated in Flint by mapping them onto 3D morphological data. There is also a Matlab program that extracts 3D objects from medical images, and there are various other programs as well.

When Physiome.jp was first established, models were written in ISML. In 2010, the model description language was extended from ISML to PHML, and all models in the database were translated into PHML. Databases at Physiome.jp were fully renovated at the end of 2013, and updated again in 2015, to keep pace with advances in physiome and systems biology. In the update, we implemented additional features of the databases, which can be used as information infrastructure to facilitate collaborative mode development. In this article, we introduce Physiome.jp databases, focusing on newly developed features, including linkage with PhysioDesigner and Flint.

## 2. Materials and methods

**Figure 1:**
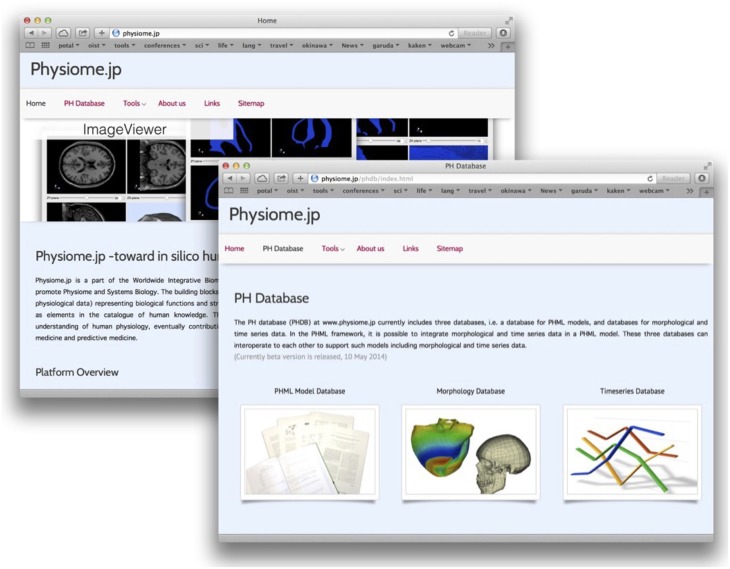
**Snapshots of the Physiome.jp portal site (left back), and the PH Database top site (right front)**. Three databases and tools for supporting research in physiome and systems biology are available at http://physiome.jp. The PH Database contains databases of PHML models, and morphological and time-series data.

Databases at Physiome.jp (http://physiome.jp), collectively denominated the PH Database, comprise three databases that manage PHML mathematical models, morphological data, and time-series data (Figure 1). The interface and framework of the PH Database were written in Python using a high-level Python web framework called django (https://www.djangoproject.com). The backend databases are implemented with MySQL (http://www.mysql.com).

The most significant new features of the PH Database include the following items (explained in the Results Section):

- The three databases cooperate with each other.
- A tree-structure of models can be viewed on a web browser.
- User accounts are managed.
- Sharing level of the models can be controlled.
- Update histories of models are logged.
- Sets of parameter values for a model can be managed.
- Interoperation with modeling and simulation tools.
- Interoperation with the Garuda platform.

### 2.1. Supported file formats

The model database accepts models written in PHML. In PHML, physiological structures and functions are represented as modules. Each module is characterized by physical quantities that define dynamic variables or constants. A set of several modules can be treated as one module located in a higher level concept; hence, a model is represented as an aggregate of modules forming a tree structure. For example, an aggregate of modules representing cells can be treated as one module representing a tissue. On the other hand, it is possible to express and define quantitative (functional) interaction between modules as a network across the hierarchical structure. These two methods of model representation enable flexible modeling.

Additionally, the model database can accept PHZ files that are used by PhysioDesigner, and are zip-compressed archive files conceptually consonant with the widely used Office Open XML (ISO/IEC 29500) or Open Document formats (ISO/IEC 26300). A PHZ file includes a PHML model and other files, such as morphological and time-series data files used in the model, as well as metadata describing layout information for modules on the PhysioDesigner canvas. In the case of SBML-PHML hybrid models that include both SBML and PHML for model description (see below and Asai et al., 2014), the imported SBML model is also packed into the PHZ file.

The morphological database can manage files defining morphological data written in various formats, such as VTK, VRML, and STL formats for polygon objects, and MAT, VOL, and RAW formats for volume objects. The time-series database manages files in CSV and ISD formats. The latter is a binary format used by Flint, specifications of which are available at http://physiodesigner.org/simulation/flint/.

### 2.2. Applications interoperating with the PH database

The PH Database is constructed as a framework not only to accumulate data, but also to interoperate tools. Developing models, performing simulations, and managing models and data are the three fundamental activities of simulation-based research. Linkage between PhysioDesigner, Flint, and the PH Database form a promising framework to comprehensively support research. These tools are summarized below. To expand interoperability between databases and tools, we utilized the Garuda platform which is also introduced below.

#### 2.2.1. PhysioDesigner

PhysioDesigner provides a graphical user interface to construct mathematical models of multilevel physiological systems within the scope of PHML's capabilities (Asai et al., 2011). Modeling targets in a model are expressed as modules that are drawn as rounded rectangles on a canvas. Modules form a tree to express hierarchical structure seen in physiological functions, and form a network to express complicated functional connections. Users manipulate them with mouse operation to construct models. It is possible to incorporate morphological and time-series data to construct mathematical models. Models developed on PhysioDesigner are written in PHML or saved as PHZ files. PhysioDesigner was first developed as *insilico*IDE in 2007 (Kawazu et al., 2007), and rebranded in 2010. It is downloadable free of charge from http://physiodesigner.org.

One of the distinguishing features of PhysioDesigner is its ability to build SBML-PHML hybrid models, incorporating both SBML and PHML to describe multilevel biophysiological functions (Asai et al., 2014). Another important feature is that PhysioDesigner can extract a set of parameter values from a model, and create a parameter set file, which is written in the Physiological Hierarchy Parameter List (PHPL) markup language format, introduced at http://physiodesigner.org. PhysioDesigner can also apply a parameter set to a model to replace parameter values.

#### 2.2.2. Flint

Flint is a simulator developed with PhysioDesigner to simulate models written in PHML. It also supports SBML and SBML-PHML hybrid models. When development started in 2007, it was called *insilico*Sim (Heien et al., 2010), and then it was rebranded as Flint in 2010. In Flint, users are asked to set parameters that are required for simulation, such as duration of the simulation, time increments, and a choice of output physical quantities. Parameter scan with automatic execution of multiple simulations can be done with Flint. This is downloadable from http://physiodesigner.org/simulation/flint/.

There is a cloud-based simulation service powered by the computing core of Flint, which is called Flint K3 (Asai et al., 2012). In January 2014, version 1.0 became available at http://flintk3.org. Initially it used cloud computing resources Edubase Cloud (http://edubase.jp/cloud/), provided by the National Institute of Informatics (NII), and later moved to Academic Inter-Cloud (AIC) of NII, a bare metal academic community cloud hub based on OpenStack, in 2014 (Yokoyama and Yoshioka, 2014).

#### 2.2.3. Garuda platform

In 2009, the Garuda alliance (http://www.garuda-alliance.org/) was proposed primarily by one of the authors (HK), and the Garuda platform was developed with the aim of providing protocols and interfaces that coherently connect tools used in the fields of systems biology and physiome research (Ghosh et al., 2011, 2013). It was conceived as providing one-stop service to users. The Garuda platform acts as a hub to connect applications. PhysioDesigner and Flint already support the Garuda platform. Beside this, we have developed a client application of PH Database supporting the Garuda platform, details of which are shown in Section 3.6.

Usually to achieve intercommunication between two applications, each application needs to implement functions to exchange messages with the other application. If the number of applications increases, the number of combinations increases rapidly. Accordingly, it is difficult for a new application to join an existing applications network. The Garuda platform acts as an application network hub, and if applications implement functions to communicate with the Garuda platform (send and receive messages), a network of applications can be established that new applications can easily join. The network behaves as follows. When Application A wants to send a file to Application B, it sends the file to the Garuda platform with the destination information. Then the Garuda platform receives the file from Application A, and transfers it to Application B.

## 3. Results

### 3.1. Cooperation among databases

**Figure 2:**
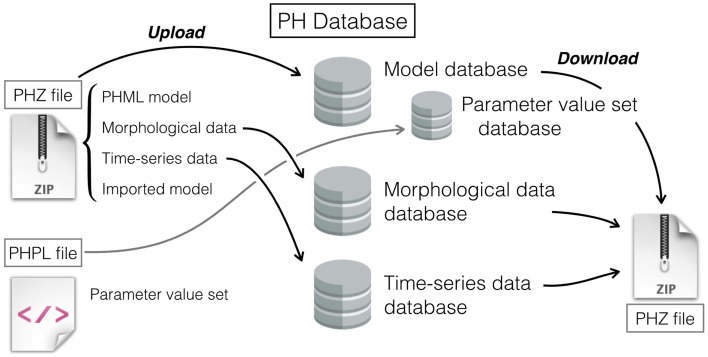
**Schematic diagram showing a process flow to upload a PHZ model and PHPL file, and to download a model**. A PHZ file may contain morphological data, time-series data, and an imported model, as well as a PHML model. Morphological and time-series data reside in each database separately, and the PHZ file is stored in the model database. Single models can be characterized by various sets of parameter values that are described in the PHPL format.

The three databases at Physiome.jp are designed to cooperate (Figure 2). This is one of the major advantages of the PH Database that, so far as we know, no other offers. Of course, each database can also be used independently.

Usually, morphological and time-series data integrated in a model reside separately from the model file. When a model is uploaded to the database, it is convenient not only to manage the model, but also the accompanying data used therein. At the PH Database, when a PHML model is uploaded, the model is examined by the server to check whether morphological or time-series data are being used. If the model includes accompanying data, the server asks the users whether they would like to register the data as well. When users register integrated data to the databases, associations between models and data are also recorded in the PH Database. This enables users to easily refer to the data from the model or vice versa, because data links are displayed when browsing models or associated data. When downloaded, a model and its data are zipped together in a compressed archive file, enabling the user to simultaneously download all files required to perform a simulation.

When a user uploads a PHZ file, data involved in the model are automatically extracted from the file and registered to the appropriate databases. The uploaded PHZ file can be downloaded as it was; hence, in this case, the original module layout is maintained because the PHZ file includes the meta-information.

A PHML file is stored as a file in the server. When a PHZ file is uploaded, a PHML file in it is extracted and stored as a PHML file, so that the server can retrieve detailed model information by parsing it using PHML APIs. However, the basic information, such as, model ID, model name, description, creator information, reference information, registered date, path to the model file, simulation result information, as well as the linkage information with morphological and time-series data, are stored in a database table to enable the PH database server to respond and to display the information quickly.

### 3.2. Online browsing of a model's tree structure

**Figure 3:**
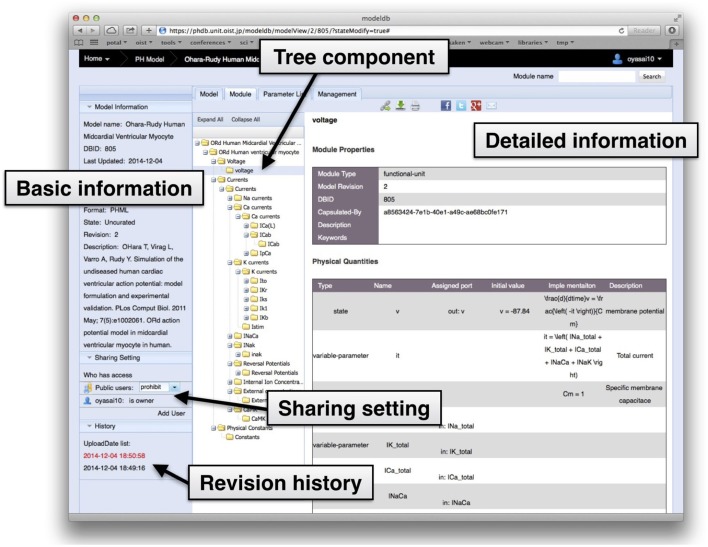
**A snapshot of the detailed information view of modules in a model on the model database**. The leftmost pane shows basic information such as model name, registrant of the model, description, and revision number. Settings for model sharing can be selected in this pane. Next to the pane is the interactive tree component, showing the tree structure of modules in the model.

In the Physiome.jp model database, an interactive component tree displays the hierarchical structure of the model, furnishes detailed information about the model, and allows users to browse modules within the model using an ordinary web browser (Figure 3). Detailed information about a selected module is shown on the right side of the tree display. Further, when an individual component is clicked, information about the physical quantity defined in the selected module is shown in the space at the right-hand side. This enables users to browse inside models, and to understand them without actually downloading them.

To display the interactive component tree of a model, the server parses it using PHML APIs, and take the module structure information. Detailed information for each module is also retrieved every time from the model.

Taking advantage of the modularity and tree-like structure, it is possible to download part of a model. When viewing detailed information about a module in the interactive tree, it is possible to click the download icon on the tool bar. If the selected module has dependent modules forming a sub-tree, all modules in the sub-tree are downloaded as well, maintaining their relationships.

### 3.3. User management and sharing

Users must create an account in Physiome.jp to login. It is also possible to login using Facebook or Google+ accounts. After logging in, users can work in a convenient, customized environment. For example, a “favorites list” function is available, to which users can add any publicly available models in order to easily access and reference those models afterward.

Databases in Physiome.jp are designed so that the sharing level of a model or dataset can be controlled by the model owner, the person who registered it. Models may be registered as public or private. Without logging in, users can see public models that are listed under the tab, Published Models. Private models can be shared with users specified by the model owner. Only the model owner and users with whom the model was shared can see the model under the tab Shared Models. This function allows the PH Database to be used as a platform to share models with development team members.

### 3.4. Update history of models

Models and data in the PH Database can be updated by the owner without removing previous versions. Every time data are updated, the revision number is incremented and previous datasets also remain accessible. It is also possible for owners to delete specific versions, if desirable. If models in the history are designated as public, users can download previous versions of those as well.

### 3.5. Management of parameter sets

It is possible for one model to reproduce different dynamics, such as healthy and pathological states, or regular firing vs burst firing neurons, by changing only the parameter values in the model without modifying the equations. In conventional databases, the only way to manage such cases is to replicate the model with different parameter values, and save the results as different models. Users can then register each model to a database. But when users need to modify formulae in the model, the same modifications have to be applied manually to all model variations with different parameter values to maintain consistency.

In contrast to the conventional databases, the model database at Physiome.jp can manage a model and parameter value sets separately (Figure 2). That permits users to register multiple PHPL files to a single model. After downloading a model, users can select specific PHPL files to download in order to obtain the intended dynamics.

### 3.6. Interoperation among applications

**Figure 4:**
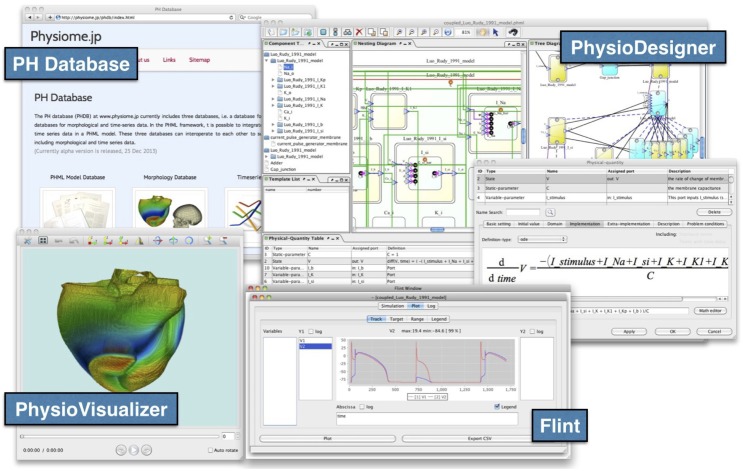
**Snapshots of PhysioDesigner, Flint, PhysioVisualizer, and the PH Database**. PhysioDesigner, a versatile platform for modeling multilevel physiological systems, can access model databases in the PH Database, and can download them onto the canvas. Flint runs simulations of models. If morphological data are integrated into the model, simulation results can be visualized with PhysioVisualizer.

The PH Database equips the REST (REpresentational State Transfer) API to accept queries from web-based and standalone applications. For example, PhysioDesigner can access the model database directly through the REST API, i.e., to search for models in the model database, and to download and expand them on the canvas. PhysioDesigner also enables users to upload models being edited on the canvas directly to the model database (Figure 4).

To perform simulations using Flint, users download models of interest from the database into PhysioDesigner, and call Flint from PhysioDesigner directly. Models are passed to Flint automatically. Flint K3 is capable of retrieving a model from the model database at Physiome.jp directly. This direct link between web-based systems is convenient for users to check the dynamics of models in the PH Database without installing Flint or PhysioDesigner on a local CPU.

**Figure 5:**
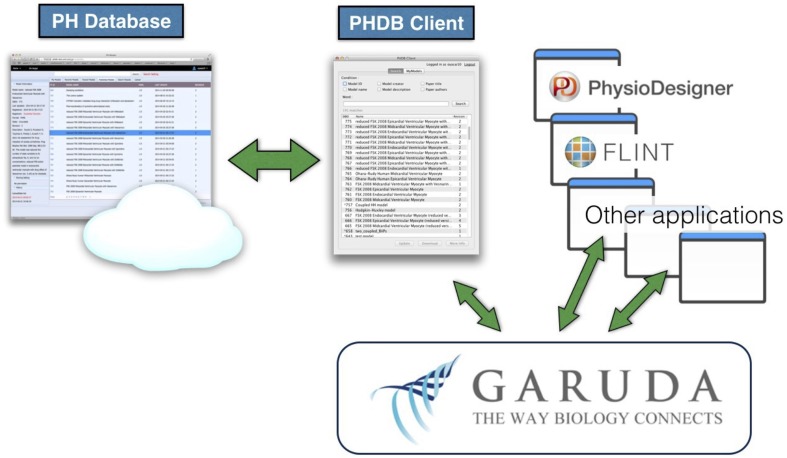
**Schematic diagram showing how the PH Database interoperates with other applications via the PHDB Client and the Garuda platform**. The PHDB Client can directly access the model database in the PH Database. At the same time, it supports the Garuda platform, which allows the PHDB Client to communicate with other Garuda-compatible applications.

To expand interoperability of the PH Database with various other applications, we utilized the Garuda platform. Because the PH Database is web-based, at present it cannot cooperate directly with the Garuda platform. Therefore, we have developed a client application for the model database that is compatible with Garuda. It accesses the model database through the REST API, acting as a standalone graphical interface for the model database, and allowing users to search and download/upload models (Figure 5). It also can exchange PHML models through the Garuda platform with other Garuda-compatible software applications. The PH Database client and the Garuda platform provide a way to connect the PH Database and any applications that could not directly interoperate. This client application can be downloaded from a portal site, Garuda Gateway, which collects applications supporting the Garuda platform: http://gateway.garuda-alliance.org.

Furthermore, it is also possible to utilize emerging web/network technologies, such as Facebook (http://facebook.com), Twitter (http://twitter.com), Evernote (http://evernote.com), and Dropbox (http://www.dropbox.com). They provide an OAuth authorization framework (http://tools.ietf.org/html/rfc6749), allowing applications to access their APIs. First, Physiome.jp utilizes their APIs for user authentication. Utilizing accounts that users already have on those network services is not only convenient for users, but the database site manager can also reduce security risks of personal information leaks. Second, their APIs enable applications to share models and data through their network services. With the PH Database, it is possible to upload a model to, for example, a closed group on Facebook or Google+, in which a discussion of the model is being conducted.

## 4. Discussion

### 4.1. Number of users access

Using the update to the Google Analytics service of late 2013, Physiome.jp was equipped with a counter in order to track site visits. Since 1 December 2013, the number of visitors was approximately 200 per month. In May 2015, when the latest system of the PH Database was released, the number of visitors jumped to about 1000 per month, and afterward stabilized at about 700 per month (the last count for this manuscript was on 28 July 2015). One reason for the increased site use since May 2015 appears to be the newly implemented interoperability with standalone applications, which leads application users to the site, or allows users to access the PH Database via web browsers.

### 4.2. Comparison with other databases

There are several databases of model description languages, such as, BioModels (Le Novère et al., 2006; Chelliah et al., 2014) and the CellML Repository (Lloyd et al., 2008) of models written in SBML and CellML. We examined differences and similarities of the PH Database and these two pioneering efforts. These are summarized in Table 1.

**Table 1:** **Comparison of functions among three databases, the PH Database, BioModels, and the CellML Repository**.

	**PH database**	**BioModels**	**CellML repository**
URL	http://physiome.jp/phdb/	https://www.ebi.ac.uk/biomodels-main/	https://models.physiomeproject.org/
Supporting file formats	PHML^*a^, PHPL	SBML, CellML	CellML, FieldML
Cooperation among databases of models, morphological data, and timeseries data	✓		
Browse model contents with interactive tree view	✓		
User account / management	✓		✓
Public/Private level for model sharing	✓		✓
Model revision history	✓		✓
Sets of parameter values for a model	✓		
APIs for direct access from applications	✓	✓	✓
On-line simulation	Flint K3	BioModels Online, JWS Online, Flint K3, Online Copasi	Online Copasi
Model curation		✓	✓
Ontology-based search		✓	✓

*a *PHML has the ability to import a whole SBML model into modules, and employs SBML-PHML hybrid modeling to describe hierarchical biophysiological functions (Asai et al., 2014). However, the PH Database does not use SBML directly; hence, SBML is not listed in the table*.

*Functions supported by a given database are designated with a ✓ mark*.

The PH Database accepts files written in PHML and PHPL formats. PHML has the capacity to import a whole SBML model into modules, and create a SBML-PHML hybrid model to describe hierarchical biophysiological functions (Asai et al., 2014). When users upload such a hybrid model, the PH Database does not treat SBML directly, but considers it as a part of a PHML model; hence, SBML is not listed in the table.

Functions to browse model hierarchical structure in an interactive tree view and to manage parameter value sets for every model are unique to the PH Database. Interoperation among three databases of models, morphological data and time-series data is another advantage of the PH Database. The CellML Repository includes a database of FieldML, which can be used to represent dynamic 3D geometry and solution fields from computational models of physiological functions (Britten et al., 2013). However, repositories of CellML models and FieldML do not interoperate as implemented in the PH Database. User management, model sharing level control (public/private), and model revision history are available in the PH Database and CellML Repository. All three databases equip APIs to receive queries from outside. In all cases, servers return JavaScript Object Notation (JSON) objects to queries, so that clients can process the answer easily with existing JSON decoders. APIs of the PH Database and CellML Repository are implemented based on the REST architecture, and that of BioModels is based on SOAP architecture. The PH Database and CellML Repository provide APIs to login to a private workspace. After logging in, users can access to private models.

Model curation, which is established in the BioModels and CellML Repository, is an important process to operate and maintain the model databases (discussed in the following section). The other issue that must be considered from the view point of user experience, as well as database management, is the ontology-based search function. BioModels makes ontology-based searching available to all users. In the CellML Repository, ontology-based searching is available only to registered users. In the PH Database, this has not been enabled yet; however, it is under development, not only for model searches, but also to link other databases.

### 4.3. Reproduction of simulation results

Models stored in the PH Database and other databases, such as BioModels and CellML Repository (those written in model description languages such as PHML, SBML, and CellML), define mathematically described dynamics. Simulation results can differ depending on numerical integration methods employed. Reproduction of simulation experiment results by different users and software tools must be ensured. To assure reproducibility, Minimum Information About a Simulation Experiment (MIASE) has been proposed as a guideline (Waltemath et al., 2011a). In order to encode information required by MIASE in a computer-readable exchange format, the Simulation Experiment Description Markup Language (SED-ML) (Waltemath et al., 2011b) was developed.

**Figure 6:**
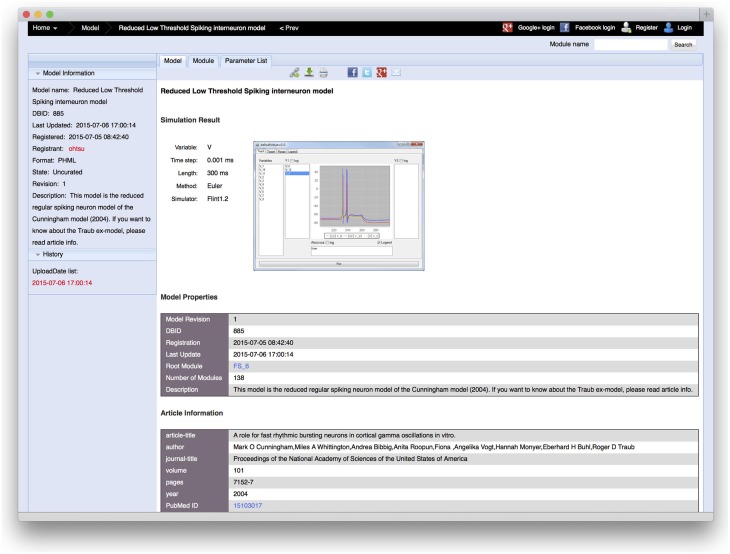
**A detailed information view of a model, in which an example of a simulation result and simulation conditions, such as the variable name to be plotted, time step, simulation duration, numerical algorithm, and tool used for the simulation are shown**. When users upload a model, the server requests the user to provide this information. Beside that, the model registration date, description of the model, article information in which the model was proposed, and model creator information, which can be described in the model, are shown.

In the PH Database, one or more examples of simulation results can be uploaded to the database with a model. When a user uploads a model, the server requests the user to provide figures of simulation results, the variables to be plotted, time steps, simulation duration, numerical algorithms, and tools used for the simulation, which are shown in the detailed view of the model (Figure 6). These adhere to MIASE guidelines, based on which, any user can reproduce the simulation results.

When a model is registered with multiple parameter sets written in PHPL, examples of simulation results and simulation conditions must be provided for each case. SED-ML intended to describe simulation conditions, including parameter values to be used in simulations; hence, it should be appropriate for this application. However, it was developed primarily by the systems biology community, so compatibility of SED-ML with PHML, which is an emerging language outside the systems biology community, is less complete than with SBML and CellML. We therefore adopted the PHPL format just to describe parameter value sets of variables in a PHML model. Still, efforts to apply SED-ML and its features to our use case is an ongoing focus to ensure robustness and extensibility of the simulation platform. We have already started to employ SED-ML in the Flint backend. When Flint sends a simulation request to Flint K3 to run simulations on cloud resources rather than on a desktop machine, it sends a SED-ML file with the model.

### 4.4. Challenges on data curation

Maintaining high quality models in the databases is a significant challenge. The Physiome.jp model database implements a systematic scheme to support a curation process similar to that adopted by BioModels at EMBL-EBI, which assures certain aspects of model quality.

After logging into the PH Database, a user who registers a model can send a curation request to model curators at Physiome.jp. The model must be open to the public. On the curatorial side, curators check whether the model meets certain criteria, i.e., whether the model can be simulated by Flint, and whether the simulation results are consistent with results published in the paper on which the model was based. Physiome.jp does not judge the model's scientific correctness. That must be undertaken in the research publication phase.

After curation is completed, the user is informed of the curation outcome via the PH Database interface. The user who registered the model can view the result of curation in the display after logging in. All curated models are shown in the list of published models with the “curated” label, so that any users searching can find them. A model for which a curation request was rejected cannot be resubmitted unless it has been updated before another curation request. To implement this process, it is necessary to have enough curators at Physiome.jp. As there are currently insufficient curators, the process has been temporarily suspended.

In the case of model curation in BioModels, models must meet Minimum Information Requested In the Annotation of biochemical Models (MIRIAM) (Le Novère et al., 2005). MIRIAM establishes guidelines to encode and annotate models in machine-readable form to facilitate model reuse with enough information about the associated reference descriptions and the biological phenomena that the model represents. The PH Database does not explicitly test uploaded models against MIRIAM. However, PHML specification includes most of requirements from MIRIAM, except for a declaration about the terms of distribution. The PH Database controls the publication level of models, and when users upload a model, they select public or private publication. This setting corresponds to the terms of distribution, and makes the model MIRIAM-compliant.

### Conflict of interest statement

The authors declare that the research was conducted in the absence of any commercial or financial relationships that could be construed as a potential conflict of interest.
